# MIS-C pathogenesis: immune dysregulation & viral triggers

**DOI:** 10.3389/fimmu.2025.1624963

**Published:** 2025-11-26

**Authors:** Tiantian Xu, Jiamin Zhang, Xiangyuan Hou, Xinyu Xie, Junye Qi, Changbing Wang, Yuqing Yan, Lu Kuang, Bing Zhu

**Affiliations:** 1 Center Laboratory, Guangzhou Women and Children’s Medical Center, Guangzhou Medical University, Guangzhou, China; 2 Guangzhou Medical University, Guangzhou, China; 3 Department of Respiration, Guangzhou Women and Children’s Medical Centre, Guangzhou Medical University, Guangzhou, China

**Keywords:** children, MIS-C, immune dysregulation, viral triggers, SARS-CoV-2

## Abstract

Multisystem Inflammatory Syndrome in Children (MIS-C) is a serious condition emerging during the COVID-19 pandemic, strongly associated with prior SARS-CoV-2 infection. Characterized by systemic inflammation affecting multiple organs, MIS-C presents a complex clinical picture including fever, gastrointestinal distress, cardiac dysfunction, and neurological manifestations. Although its exact pathogenesis remains incompletely understood, immune dysregulation is recognized as a central mechanism. This review examines current understanding of MIS-C pathogenesis, focusing on immune dysfunction and viral triggers, particularly SARS-CoV-2. We analyze both innate and adaptive immune responses, cytokine storm dynamics, molecular mimicry, and virus-induced inflammatory cascades. Additionally, we discuss potential immunomodulatory therapeutic strategies and identify future research directions to improve MIS-C management and treatment outcomes.

## 1 Introduction

The COVID-19 pandemic has highlighted multisystem inflammatory syndrome in children (MIS-C), a severe condition typically emerging 2–6 weeks after SARS-CoV-2 infection (1–3). While rare hyperinflammatory responses were previously documented in pediatric cases of Middle East Respiratory Syndrome (MERS) and Severe Acute Respiratory Syndrome (SARS), MIS-C demonstrates a distinct clinical characterized by more pronounced gastrointestinal and mucocutaneous involvement. MIS-C presents with fever, abdominal pain, and rash, and cardiovascular manifestations, posing substantial diagnostic challenges (4–7). Its pathophysiology involves dysregulated hyperinflammatory responses to viral exposure, evidenced by elevated inflammatory markers including C-reactive protein (CRP) and interleukin-6 (IL-6) (8–10). This immune dysregulation likely stems from genetic predispositions interacting with environmental triggers, particularly viral infections. The immune activation pattern shows partial overlap with Kawasaki Disease (KD) but demonstrates distinct cytokine profiles and epidemiological characteristics (11, 12). The temporal delay between SARS-CoV-2 infection and MIS-C onset supports classification as a post-infectious syndrome rather than direct viral pathology, a crucial distinction guiding therapeutic strategies (6, 13–15).

Clinical management remains challenging without standardized protocols. Current guidelines recommend immunomodulatory therapies including intravenous immunoglobulin (IVIG) and corticosteroids, though variable treatment responses necessitate further investigation through controlled trials (16–18). Emerging research focuses on identifying predictive biomarkers to enable personalized treatment approaches (10, 19–21).

Understanding the interplay between viral triggers and immune dysregulation remains essential for elucidating MIS-C pathogenesis. Continued investigation into immunological mechanisms and therapeutic interventions will be critical for improving outcomes and preparing for future pediatric health emergencies.

## 2 Clinical manifestations and diagnostic criteria of MIS-C

### 2.1 Characteristic symptom presentations

MIS-C is a severe inflammatory complication of SARS-CoV-2 infection, with clinical features overlapping KD (19, 22, 23). Key symptoms include persistent fever (present in nearly all cases), rash, conjunctivitis, and gastrointestinal issues such as abdominal pain, vomiting, and diarrhea. Cardiovascular manifestations (e.g., shock) and neurological symptoms (e.g., altered mental status or seizures) may also occur, complicating diagnosis (5, 24, 25). Elevated inflammatory markers—including C-reactive protein (CRP), ferritin, and D-dimer—are common and aid in assessing severity (26–28). Some children exhibit acute respiratory symptoms, potentially leading to misdiagnosis as primary respiratory infections (29).

Epidemiological data reveal significant disparities in MIS-C prevalence across populations. Incidence rates are higher among Hispanic/Latino and non-Hispanic Black children compared to non-Hispanic White children, with variations linked to genetic, socioeconomic, and geographic factors (30–32). Age and gender also influence risk, with peak incidence in children aged 5–12 years and a male predominance (ratio ~1.5:1) (33). These differences underscore the need for population-specific clinical awareness.

**Figure 1:**
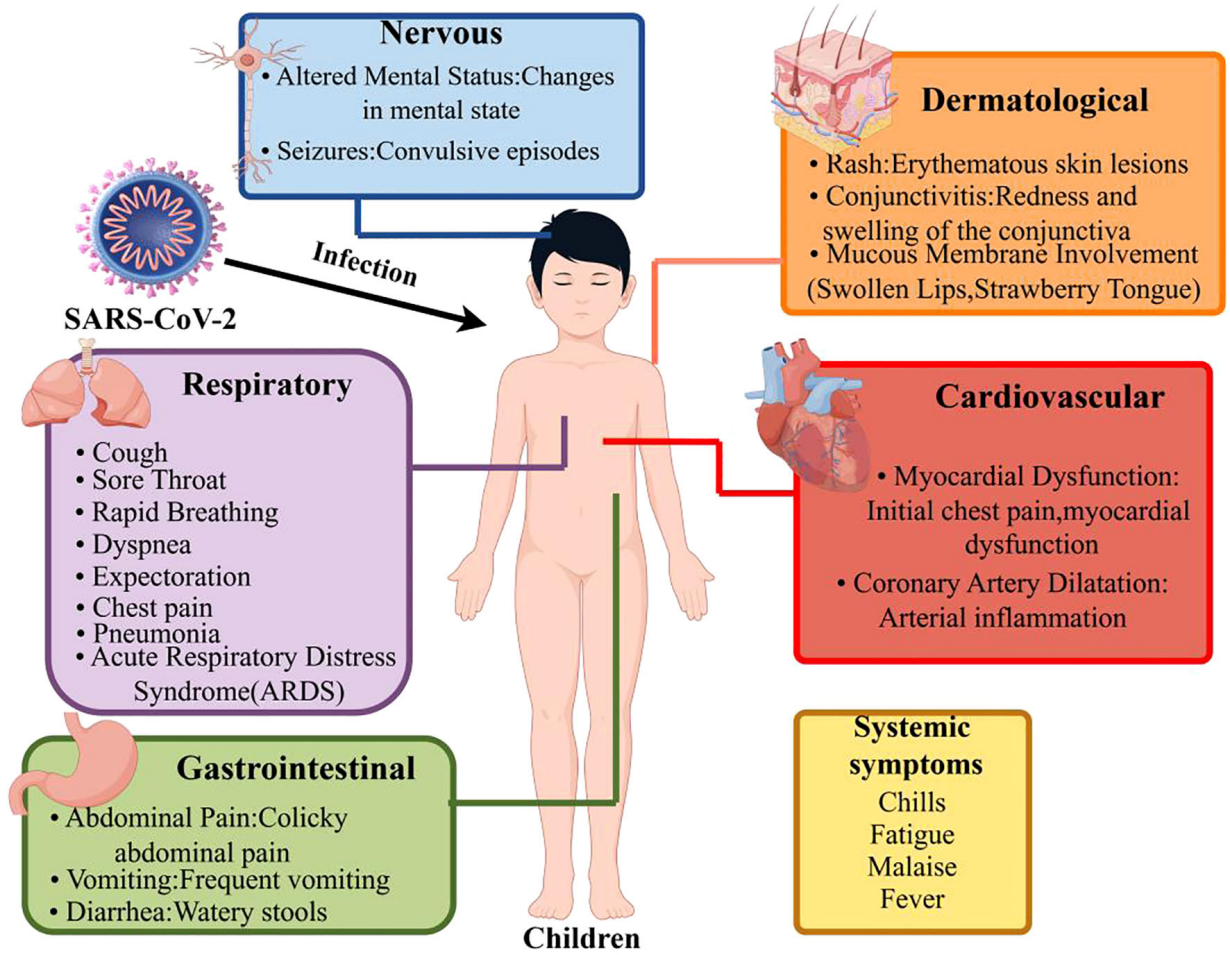
Symptom characteristics simulation chart of MIS-C following SARS-CoV-2 infection (By Figdraw). This schematic summarizes the multisystem clinical features observed in children with MIS-C following SARS-CoV-2 infection. The characteristic symptoms involve multiple organ systems: Cardiovascular: Myocardial dysfunction (e.g., chest pain), coronary artery dilatation, and arterial inflammation. Gastrointestinal System: Abdominal pain (often colicky), vomiting, and diarrhea (watery stools). Skin and Mucous Membranes: Erythematous rash, non-purulent conjunctivitis, and mucosal involvement. Neurological System: Altered mental status and seizures. Respiratory System: Respiratory distress, cough, and pneumonia. Systemic Symptoms: Persistent fever, fatigue, myalgia, and chills. This comprehensive depiction highlights the systemic hyperinflammation and heterogeneous presentation in MIS-C, which distinguishes it from other pediatric inflammatory conditions.

Given the symptom overlap with other inflammatory conditions, high suspicion and comprehensive assessment are essential for accurate differentiation. A schematic of MIS-C symptom characteristics is shown in **Figure 1**.

### 2.2 Diagnostic approaches

Diagnostic MIS-C relies on clinical evaluation and laboratory testing, based on criteria from the Centers for Disease Control and Prevention (CDC) and the World Health Organization (WHO) (34–36). Key requirements include fever lasting ≥ 24 hours, elevated inflammatory markers (e.g., CRP, erythrocyte sedimentation rate, or ferritin), and involvement of ≥2 organ systems (e.g., cardiovascular, gastrointestinal, or neurological). Providers must exclude alternative causes, such as active infections or other inflammatory syndromes.

Early identification is critical for initiating treatments like intravenous immunoglobulin (IVIG) and corticosteroids. The diagnostic process should account for demographic variations; for instance, higher prevalence in certain populations may warrant adjusted thresholds for inflammatory markers (30, 37). MIS-C typically presents as multi-system inflammation weeks post-SARS-CoV-2 infection, and confirmation involves characteristic markers and organ involvement, as summarized in **Table 1**.

**Table 1:** Diagnostic criteria and key points of MIS-C.

Diagnostic criteria	Description	Ref.
Fever	Fever is a common symptom of MIS-C, with no specific duration required. Any subjective or documented fever (≥38°C) is sufficient for inclusion.	(19) (22),
Age	MIS-C primarily occurs in individuals under 21 years of age, typically 2-6 weeks after SARS-CoV-2 infection. The average age of MIS-C patients is around 10 years.	(91)
Multi-Organ Involvement	MIS-C involves multiple organ systems, including cardiovascular, gastrointestinal, hematologic, respiratory, and neurological systems. Cardiac dysfunction, such as reduced left ventricular ejection fraction (<55%) or coronary artery dilation, is common.	(19) (39),
Clinical Severity	MIS-C presents with fever, gastrointestinal symptoms, cardiovascular issues, rash, conjunctivitis, abdominal pain, vomiting, diarrhea, and signs of shock. Neurological symptoms such as altered mental status and seizures may also occur.	(19) (22),
Systemic Inflammation	Elevated levels of inflammatory markers such as C-reactive protein (CRP ≥3.0 mg/dL), ferritin, and D-dimer are common in MIS-C patients. The CRP threshold of ≥3.0 mg/dL is required to indicate systemic inflammation.	(25) (26),
Epidemiologic Linkage	MIS-C is epidemiologically linked to SARS-CoV-2 infection, typically occurring 2-6 weeks after the infection.	(19) (30),
Laboratory Evidence of SARS-CoV-2	Laboratory evidence of SARS-CoV-2 infection is typically required, such as positive nucleic acid amplification tests (NAAT), antigen tests, or antibodies. Testing must occur within 60 days before or during hospitalization or after hospitalization.	(19) (31),
Exclusion of Other Diagnoses	The diagnosis of MIS-C requires the exclusion of other possible causes, such as active infections or other inflammatory conditions, including Kawasaki disease and toxic shock syndrome.	(19) (30),

CRP, C-reactive protein; MIS-C, multisystem inflammatory syndrome in children; SARS CoV-2, severe acute respiratory syndrome coronavirus 2.

### 2.3 Laboratory and imaging evaluations

Laboratory tests are crucial for diagnosing and managing MIS-C. Common findings from these tests often show elevated inflammatory markers, including CRP, procalcitonin, ferritin, and D-dimer, alongside conditions like lymphopenia and thrombocytopenia (38, 39). Additionally, cardiac biomarkers such as troponin and B-type natriuretic peptide (BNP) are frequently elevated, suggesting possible heart involvement (40). Imaging studies, especially echocardiography, are vital for evaluating cardiac function and detecting complications like coronary artery dilation or aneurysms, which pose significant risks in MIS-C cases (41). Chest imaging may also be necessary to check for lung involvement. By integrating clinical observations, laboratory results, and imaging findings, healthcare providers can conduct a thorough assessment of the child’s health, which is essential for making informed treatment decisions and monitoring for potential complications. In summary, effectively managing MIS-C requires a comprehensive approach that combines symptom evaluation, laboratory testing, and imaging studies to ensure prompt and effective care for this serious condition.

## 3 Abnormal immune responses

### 3.1 Mechanisms of autoimmunity and viral cross-reactivity

Autoimmunity arises from genetic, environmental, and immunological imbalances that disrupt immune tolerance, triggering activation of self-reactive T and B cells (42). These cells produce autoantibodies and pro-inflammatory cytokines, driving tissue injury and inflammation (43). Infections can exacerbate such responses, as seen in MIS-C, where viral epitopes induce cross-reactivity:

EBV as Primary Driver: The EBV nuclear antigen 2-derived peptide TVFYNIPPMPL is a dominant cross-reactive epitope. TCRVβ21.3+ CD8+ T cells (expanded in 82% of MIS-C patients) show 3.1-fold stronger reactivity to this EBV epitope than non-EBV antigens. Single-cell TCR sequencing confirms MIS-C T-cell repertoires cluster with EBV-specific (not SARS-CoV-2-specific) TCRs, indicating EBV-directed responses are central to cross-reactivity (44).

SARS-CoV-2 as Potential Initiator: Structural models suggested SARS-CoV-2 spike protein superantigen-like activity, but functional studies show limited T-cell activation by SARS-CoV-2 peptides. Pre-pandemic MIS-C cases indicate spike protein is non-essential. Instead, SARS-CoV-2 may dysregulate TGFβ, impairing TCRVβ21.3+ T-cell cytotoxicity against EBV-infected B cells, promoting viral persistence and inflammation (45).

HLA-Mediated Amplification: HLA risk alleles (e.g., HLA-DRB101, HLA-DQB105), present in 19.4% - 23% of MIS-C patients (absent in controls) and enhance presentation of EBV/host molecular mimics, analogous to HLA-B27 in ankylosing spondylitis (46).

Autoantibody Pathogenesis: Autoantibodies targeting endothelial, myocardial, and gastrointestinal antigens contribute to multi-organ damage. Recent studies link specific autoantibody profiles to cardiac dysfunction in MIS-C, highlighting their role in disease severity (47).

Supporting evidence includes 79.7% EBV seropositivity in MIS-C patients (vs. 56% controls) with elevated anti-EBNA2 IgA, and organ-specific inflammation aligning with EBV/host antigen mimicrysites (44). Infections trigger autoreactive lymphocytes via molecular mimicry or epitope spreading, while inflammatory cytokines (e.g., IL-6) further drive the autoimmune, as observed in lupus and rheumatoid arthritis. Understanding these mechanisms—viral cross-reactivity, HLA restriction, and autoantibody effects—is critical for targeted therapies to restore immune tolerance (46).

### 3.2 Inflammatory cytokine expression

Inflammatory cytokines balance protective immunity and harmful inflammation (48–50). Autoimmune diseases often disrupt this balance, elevating pro-inflammatory cytokines (e.g., TNF-α, IL-6, IL-1β) that correlate with disease severity (51–54). These cytokines sustain the inflammatory cycle by shaping the immune environment and driving immune cell activation and differentiation. For example, in rheumatoid arthritis, persistent cytokine exposure promotes joint damage and systemic complications (55–57). Consequently, targeting cytokine signaling pathways represents a promising therapeutic strategy to restore pro-inflammatory and anti-inflammatory balance in autoimmune diseases.

### 3.3 Immune cell activation status

Immune cell activation status critically shapes immune responses and significantly influences autoimmune disease onset and progression (58–63). In autoimmune diseases, T cells, B cells, and macrophages often exhibit hyperactivation. For example, CD4+ T helper cells differentiate into pro-inflammatory Th1 and Th17 subsets, releasing cytokines that amplify inflammation and tissue injury. Similarly, B cells produce autoantibodies that further propagate autoimmune pathology. This dysregulation disrupts immune tolerance, activating autoreactive lymphocytes (64–67). Meanwhile, regulatory T cells (Tregs) frequently show impaired function, failing to control inflammatory responses (68–72). Understanding these cellular activation patterns is therefore essential for developing targeted immunotherapies to reestablish immune equilibrium and prevent tissue damage.

## 4 Virus trigger mechanisms

### 4.1 Immune response to SARS-CoV-2 infection

The immune response to SARS-CoV-2 involves complex innate and adaptive interactions (73–75). Infection triggers innate immunity via pattern recognition receptors (PRRs) like Toll-like receptors (TLRs), which detect viral components (76, 77). This recognition induces type I and III interferons, creating an antiviral state. However, SARS-CoV-2 evades strong interferon responses compared to SARS-CoV, despite efficient replication (78, 79).

Such evasion delays adaptive immunity, leading to low-affinity antibodies and T-cell hyperactivation that worsen disease outcomes. Additionally, immune dysregulation causes cytokine storms with pro-inflammatory cytokines like IL-6, IL-1β, and TNF-α, resulting in tissue damage and respiratory issues (80–83).

**Figure 2:**
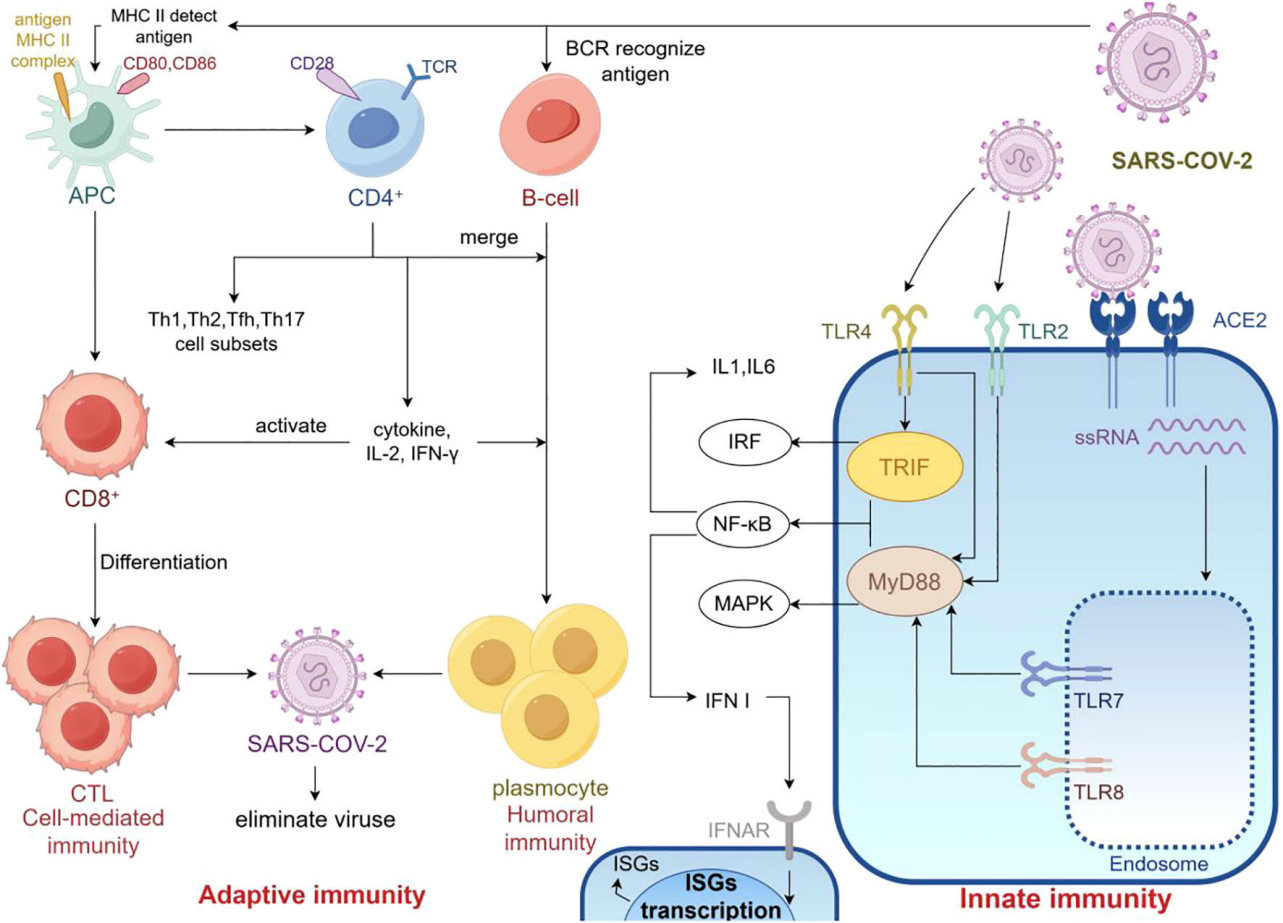
Immunological mechanisms in MIS-C (By Figdraw). MIS-C is a severe inflammatory syndrome associated with infection, characterized by aberrant activation of both innate and adaptive immunity. Innate immunity: TLR2 and TLR4 trigger pro-inflammatory cytokine release; TLR7 and TLR8 detect viral ssRNA, activating type I interferons via the MyD88 pathway; type I interferons bind to IFNAR receptor, activating JAK-STAT signaling and inducing interferon-stimulated genes (ISGs) to inhibit viral replication. Adaptive immunity: Antigen-presenting cells (APCs) process viral antigens and present them to naïve CD4^+^ and CD8^+^ T cells; activated CD4^+^ T cells differentiate into subsets such as Th1, secreting cytokines to coordinate immune responses; CD8^+^ T cells, with CD4^+^ T cell help, become cytotoxic T lymphocytes that directly kill infected cells; B cells, activated by CD4^+^ T cells, differentiate into plasma cells that secrete IgM and IgG antibodies through class switching; memory B and T cells are formed to provide long-term immunity. This dysregulated immune activation contributes to the hyperinflammation seen in MIS-C.

As shown in **Figure 2**, TLR and RLR hyperactivation drives excessive cytokine production, triggering systemic inflammation and multi-organ injury. Acute infection involves innate cell activation and lymphopenia, which normalizes during recovery. In contrast, MIS-C exhibits delayed, exaggerated immune responses with elevated inflammatory markers and tissue damage.

Epidemiological studies further reveal that MIS-C cases typically peak 2–6 weeks after community surges of SARS-CoV-2 infection, with incidence rates proportional to transmission levels (84–86).This temporal clustering, with cases surging 2–6 weeks post-infection waves, underscores the importance of monitoring infection spread for early MIS-C detection. For instance, surveillance data indicate a direct correlation between regional COVID-19 incidence and subsequent MIS-C outbreaks, highlighting the need for proactive surveillance to detect early signs of the syndrome (87, 88).

### 4.2 Effects of viral variants

The emergence of viral variants has important implications for how SARS-CoV-2 causes disease and how effective public health measures can be (89, 90). Variants like Delta and Omicron have shown increased transmissibility and the ability to evade immune responses, which complicates how the body reacts to previous infections or vaccinations (91, 92). For example, spike protein mutations in the receptor-binding domain (RBD) enhance ACE2 binding, facilitating host cell entry (93, 94). Pre-existing immunity from other coronaviruses may impair neutralizing antibodies against variants, potentially exacerbating disease (95, 96). Variants’ ability to partially escape antibodies challenges vaccine efficacy, highlighting the need for updated vaccines. Ongoing surveillance is crucial to understand impacts on spread, severity, and vaccine effectiveness. These variants may also influence MIS-C incidence proportionally to infection waves, as observed in variant-specific outbreaks (97, 98).

### 4.3 Virus-induced immune hyperreactivity

Virus-induced airway hyperreactivity, especially from respiratory infections, poses a significant risk to children (99, 100). For instance, infections caused by respiratory syncytial virus (RSV) are linked to a higher likelihood of developing asthma and other chronic respiratory issues later in life (101). The causes of this hyperreactivity are complex, including direct viral effects and resulting immune imbalances. After an RSV infection, airway hyperreactivity notably increases, likely resulting from ongoing inflammation, disruption of the airway lining, and release of inflammatory substances. Furthermore, the interaction between viral infections and the body’s immune response can induce a state of heightened airway sensitivity. This increased sensitivity makes the airways more prone to future infections and allergic reactions. This situation underscores the need to better understand the long-term effects of viral infections on respiratory health, particularly in at-risk groups such as infants and young children. Addressing these challenges through preventive strategies, including vaccinations and early interventions, is crucial to reducing the incidence of chronic respiratory diseases associated with viral infections.

### 4.4 Comparative viral pathogenesis in MIS-C

**Figure 3:**
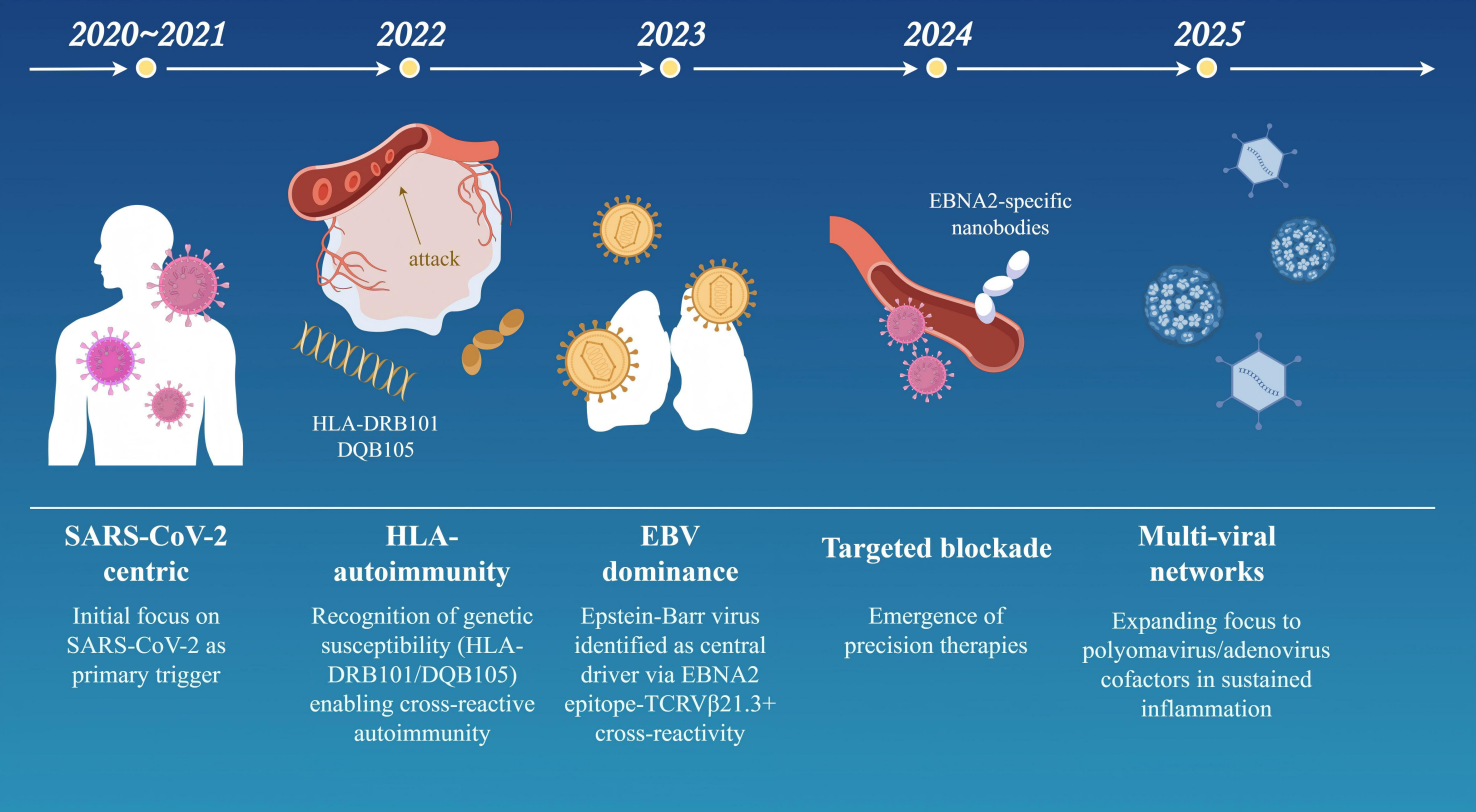
Conceptual evolution of MIS-C pathogenesis (2020–2025) (By Figdraw). This timeline model outlines key milestones in the understanding of MIS-C pathogenesis over a five-year period, reflecting shifts in research focus and mechanistic insights: 2020–2021 (SARS−CoV−2−centric phase): Initial emphasis on SARS−CoV−2 as the primary trigger, highlighting viral spike protein interactions and systemic cytokine storms. 2022 (HLA and autoimmunity): Recognition of genetic susceptibility linked to HLA−DRB101/DQB105 haplotypes, facilitating cross−reactive autoimmune responses. 2023 (Epstein–Barr virus dominance): Identification of Epstein–Barr virus (EBV) as a central driver, mediated by EBNA2 epitope cross−reactivity with TCR Vβ21.3+ T cells. 2024 (Targeted therapeutic blockade): Emergence of precision immunotherapies, including EBNA2−specific nanobodies designed to inhibit vascular leakage and hyperinflammation. 2025 (Multi−viral network hypothesis): Expanding evidence for co−factors such as polyomaviruses and adenoviruses in sustaining inflammatory networks and disease severity. The model underscores the progressive elucidation of synergistic viral, genetic, and immunologic factors in MIS-C.

Specific viruses trigger MIS-C through distinct immunopathological mechanisms. SARS-CoV-2 has the strongest causal link (>95% of cases), supported by temporal clustering, high seropositivity, and tissue viral RNA detection. Key mechanisms include spike protein superantigen-like activity driving TCR Vβ 21.3+ T-cell expansion and cytokine storms (elevated IL-6/IL-10/IFN-γ), plus ACE2-mediated endothelial damage via MMP-9 overexpression (20, 51). Unique features include IFN-I suppression and elevated NETosis markers. EBV shows a moderate association (15-20% of cases), often through reactivation evidenced by EBNA-IgG/VCA-IgM serology (102, 103). It contributes via LMP1-induced NF-κB hyperactivation and CD21+ B-cell depletion, exacerbating macrophage activation syndrome -like pathology with hyperferritinemia in co-infections. Elevated TGFβ in MIS-C impairs T cell cytotoxicity against EBV, leading to reactivation and hyper-inflammation (44). Adenovirus involvement is emerging (5-10% of cases), with hexon protein seropositivity linked to MIS-C (104). It triggers HSP60-mediated molecular mimicry against cardiac antigens and immune complex deposition driving complement-mediated NETosis, often with higher myocarditis incidence (troponin-I >1.0 ng/mL). Common pathways include triphasic immune dysregulation: viral endocytosis (via ACE2/LAT1), delayed IFN-I responses promoting pyroptosis, and TRAIL+ CD4+ T-cell cytotoxicity causing multi-organ damage. Genetic susceptibility (e.g., HLA-DRB1*11:01 allele, conferring 9.3× increased risk) further unifies these mechanisms. Milestones in MIS-C Pathogenesis are shown in **Figure 3**.

## 5 Comparisons with other pediatric inflammatory diseases

### 5.1 Distinguishing MIS-C from sepsis

Differentiating MIS-C from sepsis remains challenging due to overlapping features like fever, gastrointestinal distress, and cardiovascular instability. However, key distinctions exist: MIS-C patients are generally older (median age 10 years vs. 4 years for sepsis) and exhibit prolonged fever with prominent mucocutaneous and gastrointestinal symptoms. Laboratory findings further aid differentiation-sepsis often shows elevated leukocyte counts and procalcitonin, while MIS-C commonly presents with thrombocytopenia, lymphopenia, and hyperfibrinogenemia. Myocardial dysfunction is more severe in MIS-C and can be quantified using the Vasoactive Inotropic Score (VIS) (105). The MISSEP scoring system, with high sensitivity and specificity, assists clinicians in this distinction (16). This differentiation is vital as it informs appropriate treatment strategies; sepsis typically necessitates immediate antibiotic therapy, whereas the management of MIS-C may involve immunomodulatory treatments such as IVIG and corticosteroids.

### 5.2 Similarities and differences with Kawasaki disease

MIS-C and KD (KD) share clinical features (e.g., prolonged fever, rash, conjunctivitis, mucosal changes, and coronary artery complications), yet their pathogenesis and inflammatory profiles differ fundamentally. KD is a vasculitis treated with IVIG and aspirin, while MIS-C is a post-infectious hyperinflammatory syndrome triggered by SARS-CoV-2. MIS-C demonstrates distinct cytokine patterns, such as elevated CXCL9, rarely seen in KD (106, 107). Management strategies for MIS-C are evolving, potentially incorporating biologics tailored to its unique immunopathology.

### 5.3 Comparison with other virus-related diseases

MIS-C parallels other hyperinflammatory conditions like macrophage activation syndrome (MAS) and toxic shock syndrome (TSS) in its “cytokine storm” signature and multi-organ involvement (4). However, unlike typical viral infections presenting with respiratory symptoms, MIS-C manifests as systemic inflammation with potential organ failure (108). Its immune dysregulation may be exacerbated by SARS-CoV-2’s superantigen-like properties. These distinctions emphasize the need for comprehensive differential diagnosis in pediatric patients with systemic inflammation (98, 109, 110).

## 6 Future research directions and clinical applications

### 6.1 Identification of novel biomarkers

Novel biomarkers are critical for advancing MIS-C diagnosis and management. Recent studies highlight immunoglobulin G (IgG) glycosylation patterns—particularly afucosylated spike IgG—as promising biomarkers correlating with disease severity and hyperinflammatory responses in MIS-C patients. These glycan modifications may help track disease progression and therapeutic responses. Additional biomarkers involving cytokine/chemokine dysregulation (e.g., IL-6, CXCL9) and T-cell activation profiles are under investigation for early diagnosis and targeted therapy development (20, 106). Future research should prioritize validating large-scale validation of these biomarkers and assess their clinical utility.

### 6.2 Optimization of treatment strategies

Treatment optimization remains challenging due to MIS-C heterogeneity and variable treatment responses. Current immunomodulatory regimens (e.g., IVIG, corticosteroids) show inconsistent efficacy (111). Emerging strategies combine conventional therapies with novel agents like zonulin inhibitors to address intestinal barrier dysfunction and immune hyperactivation. Personalized approaches accounting for individual immune profiles and organ involvement may improve outcomes. Robust clinical trials evaluating combination therapies and biomarker-guided regimens are urgently needed to establish evidence-based guidelines.

### 6.3 Impact of vaccination on MIS-C incidence and severity

Vaccination significantly reduces MIS-C risk by modulating SARS-CoV-2-induced inflammatory responses. Multiple studies demonstrate 2-4-fold lower MIS-C incidence among vaccinated children, with attenuated severity in breakthrough cases (112–114). Proposed mechanisms include vaccine-induced neutralizing antibodies limiting viral replication and memory B-cell responses dampening hyperinflammation. Longitudinal data confirm sustained protection against MIS-C post-vaccination, supporting its public health value (115). Ongoing surveillance is essential to evaluate vaccine efficacy against emerging variants and optimize pediatric vaccination strategies.

## 7 Conclusion

Research on MIS-C has elucidated its complex pathophysiological, focusing on immune dysregulation and viral triggers. As a significant COVID-19 complication, MIS-C presents distinctive challenges in pediatric healthcare, requiring comprehensive understanding of its diverse clinical manifestations and immunological responses. The integration of multidisciplinary findings reveals that effective management necessitates collaborative approaches spanning immunology, pediatrics, and infectious disease specialties.

MIS-C’s heterogeneous clinical presentation—ranging from gastrointestinal to cardiovascular involvement—demands broad diagnostic criteria, while its hyperinflammatory nature underscores the need for targeted immunomodulatory therapies. Current advances not only improve clinical practice but also guide future research directions. Ongoing investigations into immune dysregulation mechanisms remain crucial for developing effective preventive and therapeutic strategies. For instance, biomarker discovery could enable risk stratification and personalized treatment approaches.

Furthermore, insights from MIS-C management inform our understanding of other pediatric inflammatory conditions and enhance preparedness for future emerging pathogens. This collective knowledge strengthens pandemic response capabilities and ultimately contributes to improved global child health outcomes.

## References

[1] Basu M Das SK . Clinical characteristics of paediatric hyperinflammatory syndrome in the era of corona virus disease 2019 (COVID-19). Indian J Clin Biochem. (2021) 36:404–15. doi: 10.1007/s12291-021-00963-4, PMID: 33716413 PMC7936863

[2] Bhat CS Gupta L Balasubramanian S Singh S Ramanan AV . Hyperinflammatory syndrome in children associated with COVID-19: need for awareness. Indian Pediatr. (2020) 57:929–35. doi: 10.1007/s13312-020-1997-1, PMID: 32683336 PMC7605487

[3] Filippatos F Tatsi EB Michos A . Immunology of multisystem inflammatory syndrome after COVID-19 in children: A review of the current evidence. Int J Mol Sci. (2023) 24:5711. doi: 10.3390/ijms24065711, PMID: 36982783 PMC10057510

[4] Panaro S Cattalini M . The spectrum of manifestations of severe acute respiratory syndrome-coronavirus 2 (SARS-coV2) infection in children: what we can learn from multisystem inflammatory syndrome in children (MIS-C). Front Med (Lausanne). (2021) 8:747190. doi: 10.3389/fmed.2021.747190, PMID: 34778310 PMC8581204

[5] Verdoni L Mazza A Gervasoni A Martelli L Ruggeri M Ciuffreda M . An outbreak of severe Kawasaki-like disease at the Italian epicentre of the SARS-CoV-2 epidemic: an observational cohort study. Lancet. (2020) 395:1771–8. doi: 10.1016/s0140-6736(20)31103-x, PMID: 32410760 PMC7220177

[6] Feldstein LR Rose EB Horwitz SM Collins JP Newhams MM Son MBF . Multisystem inflammatory syndrome in U.S. Children and adolescents. N Engl J Med. (2020) 383:334–46. doi: 10.1056/NEJMoa2021680, PMID: 32598831 PMC7346765

[7] Whittaker E Bamford A Kenny J Kaforou M Jones CE Shah P . Clinical characteristics of 58 children with a pediatric inflammatory multisystem syndrome temporally associated with SARS-coV-2. Jama. (2020) 324:259–69. doi: 10.1001/jama.2020.10369, PMID: 32511692 PMC7281356

[8] Eskandarian Boroujeni M Sekrecka A Antonczyk A Hassani S Sekrecki M Nowicka H . Dysregulated interferon response and immune hyperactivation in severe COVID-19: targeting STATs as a novel therapeutic strategy. Front Immunol. (2022) 13:888897. doi: 10.3389/fimmu.2022.888897, PMID: 35663932 PMC9156796

[9] Angurana SK Kumar V Nallasamy K Kumar MR Naganur S Kumar M . Clinico-laboratory profile, intensive care needs and short-term outcome of multisystem inflammatory syndrome in children (MIS-C): experience during first and second waves from north India. J Trop Pediatr. (2022) 68:fmac068. doi: 10.1093/tropej/fmac068, PMID: 36048462

[10] Gruber CN Patel RS Trachtman R Lepow L Amanat F Krammer F . Mapping systemic inflammation and antibody responses in multisystem inflammatory syndrome in children (MIS-C). Cell. (2020) 183:982–95.e14. doi: 10.1016/j.cell.2020.09.034, PMID: 32991843 PMC7489877

[11] Vaňková L Bufka J Křížková V . Pathophysiological and clinical point of view on Kawasaki disease and MIS-C. Pediatr Neonatol. (2023) 64:495–504. doi: 10.1016/j.pedneo.2023.05.002, PMID: 37453902 PMC10286520

[12] Sharma C Ganigara M Galeotti C Burns J Berganza FM Hayes DA . Multisystem inflammatory syndrome in children and Kawasaki disease: a critical comparison. Nat Rev Rheumatol. (2021) 17:731–48. doi: 10.1038/s41584-021-00709-9, PMID: 34716418 PMC8554518

[13] Piekarski F Steinbicker AU Armann JP . The multisystem inflammatory syndrome in children and its association to SARS-CoV-2. Curr Opin Anaesthesiol. (2021) 34:521–9. doi: 10.1097/aco.0000000000001024, PMID: 34052825

[14] Jiang L Tang K Levin M Irfan O Morris SK Wilson K . COVID-19 and multisystem inflammatory syndrome in children and adolescents. Lancet Infect Dis. (2020) 20:e276–e88. doi: 10.1016/s1473-3099(20)30651-4, PMID: 32818434 PMC7431129

[15] Hoste L Van Paemel R Haerynck F . Multisystem inflammatory syndrome in children related to COVID-19: a systematic review. Eur J Pediatr. (2021) 180:2019–34. doi: 10.1007/s00431-021-03993-5, PMID: 33599835 PMC7890544

[16] Henderson LA Canna SW Friedman KG Gorelik M Lapidus SK Bassiri H . American college of rheumatology clinical guidance for multisystem inflammatory syndrome in children associated with SARS-coV-2 and hyperinflammation in pediatric COVID-19: version 3. Arthritis Rheumatol. (2022) 74:e1–e20. doi: 10.1002/art.42062, PMID: 35118829 PMC9011620

[17] Patel JM . Multisystem inflammatory syndrome in children (MIS-C). Curr Allergy Asthma Rep. (2022) 22:53–60. doi: 10.1007/s11882-022-01031-4, PMID: 35314921 PMC8938222

[18] Gottlieb M Bridwell R Ravera J Long B . Multisystem inflammatory syndrome in children with COVID-19. Am J Emerg Med. (2021) 49:148–52. doi: 10.1016/j.ajem.2021.05.076, PMID: 34116467 PMC8185530

[19] La Torre F Taddio A Conti C Cattalini M . Multi-inflammatory syndrome in children (MIS-C) in 2023: is it time to forget about it? Children (Basel). (2023) 10:980. doi: 10.3390/children10060980, PMID: 37371212 PMC10297102

[20] Carter MJ Fish M Jennings A Doores KJ Wellman P Seow J . Peripheral immunophenotypes in children with multisystem inflammatory syndrome associated with SARS-CoV-2 infection. Nat Med. (2020) 26:1701–7. doi: 10.1038/s41591-020-1054-6, PMID: 32812012

[21] Rey-Jurado E Espinosa Y Astudillo C Jimena Cortés L Hormazabal J Noguera LP . Deep immunophenotyping reveals biomarkers of multisystemic inflammatory syndrome in children in a Latin American cohort. J Allergy Clin Immunol. (2022) 150:1074–85.e11. doi: 10.1016/j.jaci.2022.09.006, PMID: 36116582 PMC9476361

[22] Raffeiner B Rojatti M Tröbinger C Nailescu AM Pagani L . Multisystem inflammatory syndrome of adults (MIS-A) as delayed severe presentation of SARS-coV-2 infection: A description of two cases. J Clin Med. (2024) 13:6632. doi: 10.3390/jcm13226632, PMID: 39597775 PMC11594674

[23] Saeed S Cao J Xu J Zhang Y Zheng X Jiang L . Case Report: A case of multisystem inflammatory syndrome in an 11-year-old female after COVID-19 inactivated vaccine. Front Pediatr. (2023) 11:1068301. doi: 10.3389/fped.2023.1068301, PMID: 36865693 PMC9972091

[24] Chien TC Chien MM Liu TL Chang H Tsai ML Tseng SH . Adrenal crisis mimicking COVID-19 encephalopathy in a teenager with craniopharyngioma. Children (Basel). (2022) 9:1238. doi: 10.3390/children9081238, PMID: 36010128 PMC9406844

[25] García-Azorín D Abildúa MJA Aguirre MEE Fernández SF Moncó JCG Guijarro-Castro C . Neurological presentations of COVID-19: Findings from the Spanish Society of Neurology neuroCOVID-19 registry. J Neurol Sci. (2021) 423:117283. doi: 10.1016/j.jns.2020.117283, PMID: 33636661 PMC7749644

[26] Sana A Avneesh M . Identification of hematological and inflammatory parameters associated with disease severity in hospitalized patients of COVID-19. J Family Med Prim Care. (2022) 11:260–4. doi: 10.4103/jfmpc.jfmpc_941_21, PMID: 35309629 PMC8930131

[27] Sukrisman L Sinto R . Coagulation profile and correlation between D-dimer, inflammatory markers, and COVID-19 severity in an Indonesian national referral hospital. J Int Med Res. (2021) 49:3000605211059939. doi: 10.1177/03000605211059939, PMID: 34796762 PMC8619746

[28] Zhao Y Yin L Patel J Tang L Huang Y . The inflammatory markers of multisystem inflammatory syndrome in children (MIS-C) and adolescents associated with COVID-19: A meta-analysis. J Med Virol. (2021) 93:4358–69. doi: 10.1002/jmv.26951, PMID: 33739452 PMC8250955

[29] Shabbir A Khurshid H Khan IM . Multisystem inflammatory syndrome in neonates associated with COVID-19 in neonatal ICU of a tertiary care hospital. J Coll Physicians Surg Pak. (2024) 34:727–31. doi: 10.29271/jcpsp.2024.06.727, PMID: 38840360

[30] Godfred-Cato S Abrams JY Balachandran N Jaggi P Jones K Rostad CA . Distinguishing multisystem inflammatory syndrome in children from COVID-19, kawasaki disease and toxic shock syndrome. Pediatr Infect Dis J. (2022) 41:315–23. doi: 10.1097/inf.0000000000003449, PMID: 35093995 PMC8919949

[31] Payne AB Gilani Z Godfred-Cato S Belay ED Feldstein LR Patel MM . Incidence of multisystem inflammatory syndrome in children among US persons infected with SARS-coV-2. JAMA Netw Open. (2021) 4:e2116420. doi: 10.1001/jamanetworkopen.2021.16420, PMID: 34110391 PMC8193431

[32] Santos-Rebouças CB Piergiorge RM Dos Santos Ferreira C Seixas Zeitel R Gerber AL Rodrigues MCF . Host genetic susceptibility underlying SARS-CoV-2-associated Multisystem Inflammatory Syndrome in Brazilian Children. Mol Med. (2022) 28:153. doi: 10.1186/s10020-022-00583-5, PMID: 36510129 PMC9742658

[33] Abrams JY Godfred-Cato SE Oster ME Chow EJ Koumans EH Bryant B . Multisystem inflammatory syndrome in children associated with severe acute respiratory syndrome coronavirus 2: A systematic review. J Pediatr. (2020) 226:45–54.e1. doi: 10.1016/j.jpeds.2020.08.003, PMID: 32768466 PMC7403869

[34] Avcu G Arslan SY Soylu M Bilen NM Bal ZS Cicek C . Quantitative antibody levels against SARS-coV-2 spike protein in COVID-19 and multisystem inflammatory syndrome in children. Viral Immunol. (2022) 35:681–9. doi: 10.1089/vim.2022.0089, PMID: 36534467

[35] Kornitzer J Johnson J Yang M Pecor KW Cohen N Jiang C . A systematic review of characteristics associated with COVID-19 in children with typical presentation and with multisystem inflammatory syndrome. Int J Environ Res Public Health. (2021) 18:8269. doi: 10.3390/ijerph18168269, PMID: 34444014 PMC8394392

[36] Messiah SE Xie L Mathew MS Shaikh S Veeraswamy A Rabi A . Comparison of long-term complications of COVID-19 illness among a diverse sample of children by MIS-C status. Int J Environ Res Public Health. (2022) 19:13382. doi: 10.3390/ijerph192013382, PMID: 36293968 PMC9603408

[37] Son MBF Murray N Friedman K Young CC Newhams MM Feldstein LR . Multisystem inflammatory syndrome in children - initial therapy and outcomes. N Engl J Med. (2021) 385:23–34. doi: 10.1056/NEJMoa2102605, PMID: 34133855 PMC8220972

[38] Visa-Reñé N Rubio-Páez A Mitjans-Rubies N Paredes-Carmona F . Comparison of plasma inflammatory biomarkers between MIS-C and potentially serious infections in pediatric patients. Reumatol Clin (Engl Ed). (2024) 20:84–91. doi: 10.1016/j.reumae.2024.01.005, PMID: 38342738

[39] Lippi G Mattiuzzi C Favaloro EJ . Diagnostic value of D-dimer in differentiating multisystem inflammatory syndrome in Children (MIS-C) from Kawasaki disease: systematic literature review and meta-analysis. Diagnosis (Berl). (2024) 11:231–4. doi: 10.1515/dx-2024-0013, PMID: 38374575

[40] Yan Y Barbati ME Avgerinos ED Doganci S Lichtenberg M Jalaie H . Elevation of cardiac enzymes and B-type natriuretic peptides following venous recanalization and stenting in chronic venous obstruction. Phlebology. (2024) 39:619–28. doi: 10.1177/02683555241261321, PMID: 38862920

[41] Vukomanovic V Krasic S Prijic S Petrovic G Ninic S Popovic S . Myocardial damage in multisystem inflammatory syndrome associated with COVID-19 in children and adolescents. J Res Med Sci. (2021) 26:113. doi: 10.4103/jrms.JRMS_1195_20, PMID: 35126576 PMC8765507

[42] Chen B Sun L Zhang X . Integration of microbiome and epigenome to decipher the pathogenesis of autoimmune diseases. J Autoimmun. (2017) 83:31–42. doi: 10.1016/j.jaut.2017.03.009, PMID: 28342734

[43] Shan J Jin H Xu Y . T cell metabolism: A new perspective on th17/treg cell imbalance in systemic lupus erythematosus. Front Immunol. (2020) 11:1027. doi: 10.3389/fimmu.2020.01027, PMID: 32528480 PMC7257669

[44] Goetzke CC Massoud M Frischbutter S Guerra GM Ferreira-Gomes M Heinrich F . TGFβ links EBV to multisystem inflammatory syndrome in children. Nature. (2025) 640:762–71. doi: 10.1038/s41586-025-08697-6, PMID: 40074901 PMC12003184

[45] Cheng MH Zhang S Porritt RA Noval Rivas M Paschold L Willscher E . Superantigenic character of an insert unique to SARS-CoV-2 spike supported by skewed TCR repertoire in patients with hyperinflammation. Proc Natl Acad Sci U S A. (2020) 117:25254–62. doi: 10.1073/pnas.2010722117, PMID: 32989130 PMC7568239

[46] Porritt RA Binek A Paschold L Rivas MN McArdle A Yonker LM . The autoimmune signature of hyperinflammatory multisystem inflammatory syndrome in children. J Clin Invest. (2021) 131:e151520. doi: 10.1172/jci151520, PMID: 34437303 PMC8516454

[47] Bodansky A Mettelman RC Sabatino JJ Jr. Vazquez SE Chou J Novak T . Molecular mimicry in multisystem inflammatory syndrome in children. Nature. (2024) 632:622–9. doi: 10.1038/s41586-024-07722-4, PMID: 39112696 PMC11324515

[48] Turner MD Nedjai B Hurst T Pennington DJ . Cytokines and chemokines: At the crossroads of cell signalling and inflammatory disease. Biochim Biophys Acta. (2014) 1843:2563–82. doi: 10.1016/j.bbamcr.2014.05.014, PMID: 24892271

[49] Dinarello CA . Historical insights into cytokines. Eur J Immunol. (2007) 37 Suppl 1:S34–45. doi: 10.1002/eji.200737772, PMID: 17972343 PMC3140102

[50] Dzhambazov B . Special issue: autoimmune diseases: A swing dance of immune cells. Int J Mol Sci. (2025) 26:9365. doi: 10.3390/ijms26199365, PMID: 41096634 PMC12525393

[51] Feldmann M Maini RN Lasker Clinical Medical Research Award . TNF defined as a therapeutic target for rheumatoid arthritis and other autoimmune diseases. Nat Med. (2003) 9:1245–50. doi: 10.1038/nm939, PMID: 14520364

[52] McInnes IB Schett G . Pathogenetic insights from the treatment of rheumatoid arthritis. Lancet. (2017) 389:2328–37. doi: 10.1016/s0140-6736(17)31472-1, PMID: 28612747

[53] Jain A Bishnoi M Prajapati SK Acharya S Kapre S Singhvi G . Targeting rheumatoid arthritis: a molecular perspective on biologic therapies and clinical progress. J Biol Eng. (2025) 19:67. doi: 10.1186/s13036-025-00534-8, PMID: 40707975 PMC12291358

[54] Najm A Ferguson LD McInnes IB . Cytokine pathways driving diverse tissue pathologies in rheumatoid arthritis. Arthritis Rheumatol. (2025). doi: 10.1002/art.43376, PMID: 40922709

[55] Liu Y Wang XQ Lu JH Huang HW Qiu YH Peng YP . Treg cells mitigate inflammatory responses and symptoms via β2-AR/β-Arr2/ERK signaling in an experimental rheumatoid arthritis. Arthritis Res Ther. (2025) 27:194. doi: 10.1186/s13075-025-03659-9, PMID: 41107955 PMC12532925

[56] Li H Wu QY Teng XH Li ZP Zhu MT Gu CJ . The pathogenesis and regulatory role of HIF-1 in rheumatoid arthritis. Cent Eur J Immunol. (2023) 48:338–45. doi: 10.5114/ceji.2023.134217, PMID: 38558567 PMC10976655

[57] Livshits G Kalinkovich A . Hierarchical, imbalanced pro-inflammatory cytokine networks govern the pathogenesis of chronic arthropathies. Osteoarthritis Cartilage. (2018) 26:7–17. doi: 10.1016/j.joca.2017.10.013, PMID: 29074297

[58] Jacobelli J Buser AE Heiden DL Friedman RS . Autoimmunity in motion: Mechanisms of immune regulation and destruction revealed by *in vivo* imaging. Immunol Rev. (2022) 306:181–99. doi: 10.1111/imr.13043, PMID: 34825390 PMC9135487

[59] Zhao J Lu Q Liu Y Shi Z Hu L Zeng Z . Th17 cells in inflammatory bowel disease: cytokines, plasticity, and therapies. J Immunol Res. (2021) 2021:8816041. doi: 10.1155/2021/8816041, PMID: 33553436 PMC7846404

[60] Darouni L Tavassoli Razavi F Yazdanpanah E Orooji N Shadab A Emami A . Interleukin 15 and autoimmune disorders: pathophysiology, therapeutic potential, and clinical implications. Inflammation Res. (2025) 74:141. doi: 10.1007/s00011-025-02084-7, PMID: 41107624

[61] Song Y Li J Wu Y . Evolving understanding of autoimmune mechanisms and new therapeutic strategies of autoimmune disorders. Signal Transduct Target Ther. (2024) 9:263. doi: 10.1038/s41392-024-01952-8, PMID: 39362875 PMC11452214

[62] Zhou J Yang D Han C Dong H Wang S Li X . Inhibition of LARP4-mediated quiescence exit of naive CD4(+) T cells ameliorates autoimmune and allergic diseases. Nat BioMed Eng. (2025). doi: 10.1038/s41551-025-01514-5, PMID: 41102557

[63] DuJ ChenH YouJ HuW LiuJ LuQ . Proximity between LAG-3 and the T cell receptor guides suppression of T cell activation and autoimmunity. Cell. (2025) 188:4025–42.e20. doi: 10.1016/j.cell.2025.06.004, PMID: 40592325

[64] Yan J Mamula MJ . B and T cell tolerance and autoimmunity in autoantibody transgenic mice. Int Immunol. (2002) 14:963–71. doi: 10.1093/intimm/dxf064, PMID: 12147633

[65] Jung S Schickel JN Kern A Knapp AM Eftekhari P Da Silva S . Chronic bacterial infection activates autoreactive B cells and induces isotype switching and autoantigen-driven mutations. Eur J Immunol. (2016) 46:131–46. doi: 10.1002/eji.201545810, PMID: 26474536

[66] McQueen F . A B cell explanation for autoimmune disease: the forbidden clone returns. Postgrad Med J. (2012) 88:226–33. doi: 10.1136/postgradmedj-2011-130364, PMID: 22328279

[67] Hou Y Sun L LaFleur MW Huang L Lambden C Thakore PI . Neuropeptide signalling orchestrates T cell differentiation. Nature. (2024) 635:444–52. doi: 10.1038/s41586-024-08049-w, PMID: 39415015 PMC11951087

[68] An Haack I Derkow K Riehn M Rentinck MN Kühl AA Lehnardt S . The role of regulatory CD4 T cells in maintaining tolerance in a mouse model of autoimmune hepatitis. PloS One. (2015) 10:e0143715. doi: 10.1371/journal.pone.0143715, PMID: 26599014 PMC4658037

[69] Gouirand V Habrylo I Rosenblum MD . Regulatory T cells and inflammatory mediators in autoimmune disease. J Invest Dermatol. (2022) 142:774–80. doi: 10.1016/j.jid.2021.05.010, PMID: 34284898

[70] Hassan M Elzallat M Mohammed DM Balata M El-Maadawy WH . Exploiting regulatory T cells (Tregs): Cutting-edge therapy for autoimmune diseases. Int Immunopharmacol. (2025) 155:114624. doi: 10.1016/j.intimp.2025.114624, PMID: 40215774

[71] Cheru NT Osayame Y Sumida TS . Breaking tolerance: an update of Treg dysfunction in autoimmunity. Trends Immunol. (2025) 46:611–3. doi: 10.1016/j.it.2025.06.007, PMID: 40683778

[72] Peng YQ Wang L Tan AL Wang SJ Zou W Li X . PMEPA1-mediated treg cell impairment promotes endometrial stromal invasion via excessive PI3K/AKT signaling in endometriosis. Curr Med Sci. (2025) 45:1231–43. doi: 10.1007/s11596-025-00125-0, PMID: 41100035

[73] Balkhi MY . Mechanistic understanding of innate and adaptive immune responses in SARS-CoV-2 infection. Mol Immunol. (2021) 135:268–75. doi: 10.1016/j.molimm.2021.04.021, PMID: 33940513 PMC8084627

[74] Sievers BL Cheng MTK Csiba K Meng B Gupta RK . SARS-CoV-2 and innate immunity: the good, the bad, and the “goldilocks. Cell Mol Immunol. (2024) 21:171–83. doi: 10.1038/s41423-023-01104-y, PMID: 37985854 PMC10805730

[75] Shen J Fan J Zhao Y Jiang D Niu Z Zhang Z . Innate and adaptive immunity to SARS-CoV-2 and predisposing factors. Front Immunol. (2023) 14:1159326. doi: 10.3389/fimmu.2023.1159326, PMID: 37228604 PMC10203583

[76] Sariol A Perlman S . Lessons for COVID-19 immunity from other coronavirus infections. Immunity. (2020) 53:248–63. doi: 10.1016/j.immuni.2020.07.005, PMID: 32717182 PMC7359787

[77] Thorne LG Reuschl AK Zuliani-Alvarez L Whelan MVX Turner J Noursadeghi M . SARS-CoV-2 sensing by RIG-I and MDA5 links epithelial infection to macrophage inflammation. EMBO J. (2021) 40:e107826. doi: 10.15252/embj.2021107826, PMID: 34101213 PMC8209947

[78] Lei X Dong X Ma R Wang W Xiao X Tian Z . Activation and evasion of type I interferon responses by SARS-CoV-2. Nat Commun. (2020) 11:3810. doi: 10.1038/s41467-020-17665-9, PMID: 32733001 PMC7392898

[79] Blanco-Melo D Nilsson-Payant BE Liu WC Uhl S Hoagland D Møller R . Imbalanced host response to SARS-coV-2 drives development of COVID-19. Cell. (2020) 181:1036–45.e9. doi: 10.1016/j.cell.2020.04.026, PMID: 32416070 PMC7227586

[80] Mathew D Giles JR Baxter AE Oldridge DA Greenplate AR Wu JE . Deep immune profiling of COVID-19 patients reveals distinct immunotypes with therapeutic implications. Science. (2020) 369:eabc8511. doi: 10.1126/science.abc8511, PMID: 32669297 PMC7402624

[81] Li T Wang D Wei H Xu X . Cytokine storm and translating IL-6 biology into effective treatments for COVID-19. Front Med. (2023) 17:1080–95. doi: 10.1007/s11684-023-1044-4, PMID: 38157195

[82] Tian J Shang B Zhang J Guo Y Li M Hu Y . T cell immune evasion by SARS-CoV-2 JN.1 escapees targeting two cytotoxic T cell epitope hotspots. Nat Immunol. (2025) 26:265–78. doi: 10.1038/s41590-024-02051-0, PMID: 39875585

[83] Wang Y Bai C Hou K Zhang Z Guo M Wang X . Engineered dual-function antibody-like proteins to combat SARS-coV-2-induced immune dysregulation and inflammation. Adv Sci (Weinh). (2025) 12:e04690. doi: 10.1002/advs.202504690, PMID: 40619617 PMC12499391

[84] Versace V Ortelli P Dezi S Ferrazzoli D Alibardi A Bonini I . Co-ultramicronized palmitoylethanolamide/luteolin normalizes GABA-ergic activity and cortical plasticity in long COVID-19 syndrome. Clin Neurophysiol. (2023) 145:81–8. doi: 10.1016/j.clinph.2022.10.017, PMID: 36455453 PMC9650483

[85] Öztürk R Taşova Y Ayaz A . COVID-19: pathogenesis, genetic polymorphism, clinical features and laboratory findings. Turk J Med Sci. (2020) 50:638–57. doi: 10.3906/sag-2005-287, PMID: 32512673

[86] Giovanetti M Branda F Cella E Scarpa F Bazzani L Ciccozzi A . Epidemic history and evolution of an emerging threat of international concern, the severe acute respiratory syndrome coronavirus 2. J Med Virol. (2023) 95:e29012. doi: 10.1002/jmv.29012, PMID: 37548148

[87] Riphagen S Gomez X Gonzalez-Martinez C Wilkinson N Theocharis P . Hyperinflammatory shock in children during COVID-19 pandemic. Lancet. (2020) 395:1607–8. doi: 10.1016/s0140-6736(20)31094-1, PMID: 32386565 PMC7204765

[88] Dufort EM Koumans EH Chow EJ Rosenthal EM Muse A Rowlands J . Multisystem inflammatory syndrome in children in new york state. N Engl J Med. (2020) 383:347–58. doi: 10.1056/NEJMoa2021756, PMID: 32598830 PMC7346766

[89] López-Macías C López-Medina E Alves MB Matos ADR Hernández-Villena JV Aponte-Torres Z . Clinical characteristics, SARS-CoV-2 variants, and outcomes of adults hospitalized due to COVID-19 in Latin American countries. Clinics (Sao Paulo). (2025) 80:100648. doi: 10.1016/j.clinsp.2025.100648, PMID: 40273490 PMC12051657

[90] Chen Y Liu Q Zhou L Zhou Y Yan H Lan K . Emerging SARS-CoV-2 variants: Why, how, and what’s next? Cell Insight. (2022) 1:100029. doi: 10.1016/j.cellin.2022.100029, PMID: 37193049 PMC9057926

[91] Mohsin M Mahmud S . Omicron SARS-CoV-2 variant of concern: A review on its transmissibility, immune evasion, reinfection, and severity. Med (Baltimore). (2022) 101:e29165. doi: 10.1097/md.0000000000029165, PMID: 35583528 PMC9276130

[92] Arabi M Al-Najjar Y Sharma O Kamal I Javed A Gohil HS . Role of previous infection with SARS-CoV-2 in protecting against omicron reinfections and severe complications of COVID-19 compared to pre-omicron variants: a systematic review. BMC Infect Dis. (2023) 23:432. doi: 10.1186/s12879-023-08328-3, PMID: 37365490 PMC10294418

[93] Starr TN Greaney AJ Hilton SK Ellis D Crawford KHD Dingens AS . Deep mutational scanning of SARS-coV-2 receptor binding domain reveals constraints on folding and ACE2 binding. Cell. (2020) 182:1295–310.e20. doi: 10.1016/j.cell.2020.08.012, PMID: 32841599 PMC7418704

[94] Tian F Tong B Sun L Shi S Zheng B Wang Z . N501Y mutation of spike protein in SARS-CoV-2 strengthens its binding to receptor ACE2. Elife. (2021) 10:e69091. doi: 10.7554/eLife.69091, PMID: 34414884 PMC8455130

[95] Reynolds CJ Swadling L Gibbons JM Pade C Jensen MP Diniz MO . Discordant neutralizing antibody and T cell responses in asymptomatic and mild SARS-CoV-2 infection. Sci Immunol. (2020) 5:eabf3698. doi: 10.1126/sciimmunol.abf3698, PMID: 33361161 PMC8101131

[96] Anderson EM Goodwin EC Verma A Arevalo CP Bolton MJ Weirick ME . Seasonal human coronavirus antibodies are boosted upon SARS-CoV-2 infection but not associated with protection. Cell. (2021) 184:1858–64.e10. doi: 10.1016/j.cell.2021.02.010, PMID: 33631096 PMC7871851

[97] SARS-coV-2 B.1.1.529 (Omicron) variant - United States, december 1-8, 2021. MMWR Morb Mortal Wkly Rep. (2021) 70:1731–4. doi: 10.15585/mmwr.mm7050e1, PMID: 34914670 PMC8675659

[98] Dhaliwal M Tyagi R Malhotra P Barman P Loganathan SK Sharma J . Mechanisms of immune dysregulation in COVID-19 are different from SARS and MERS: A perspective in context of kawasaki disease and MIS-C. Front Pediatr. (2022) 10:790273. doi: 10.3389/fped.2022.790273, PMID: 35601440 PMC9119432

[99] Tian K Dangarh P Zhang H Hines CL Bush A Pybus HJ . Role of epithelial barrier function in inducing type 2 immunity following early-life viral infection. Clin Exp Allergy. (2024) 54:109–19. doi: 10.1111/cea.14425, PMID: 38011856

[100] Mistry LN Agarwal S Jaiswal H Kondkari S Mulla SA Bhandarkar SD . Human metapneumovirus: emergence, impact, and public health significance. Cureus. (2025) 17:e80964. doi: 10.7759/cureus.80964, PMID: 40255736 PMC12009631

[101] Petat H Gajdos V Angoulvant F Vidalain PO Corbet S Marguet C . High frequency of viral co-detections in acute bronchiolitis. Viruses. (2021) 13:990. doi: 10.3390/v13060990, PMID: 34073414 PMC8229544

[102] Papatriantafyllou M . A role for TGFβ and EBV in MIS-C pathogenesis. Nat Rev Rheumatol. (2025) 21:255. doi: 10.1038/s41584-025-01244-7, PMID: 40155695

[103] Liu M Brodeur KE Bledsoe JR Harris CN Joerger J Weng R . Features of hyperinflammation link the biology of Epstein-Barr virus infection and cytokine storm syndromes. J Allergy Clin Immunol. (2025) 155:1346–56.e9. doi: 10.1016/j.jaci.2024.11.029, PMID: 39622297

[104] Gençeli M Üstüntaş T Metin Akcan Ö Saylik S Ercan F Pekcan S . A new scoring in differential diagnosis: multisystem inflammatory syndrome or adenovirus infection? Turk J Med Sci. (2024) 54:1237–43. doi: 10.55730/1300-0144.5905, PMID: 39734335 PMC11673662

[105] Feldstein LR Tenforde MW Friedman KG Newhams M Rose EB Dapul H . Characteristics and outcomes of US children and adolescents with multisystem inflammatory syndrome in children (MIS-C) compared with severe acute COVID-19. Jama. (2021) 325:1074–87. doi: 10.1001/jama.2021.2091, PMID: 33625505 PMC7905703

[106] Caldarale F Giacomelli M Garrafa E Tamassia N Morreale A Poli P . Plasmacytoid dendritic cells depletion and elevation of IFN-γ Dependent chemokines CXCL9 and CXCL10 in children with multisystem inflammatory syndrome. Front Immunol. (2021) 12:654587. doi: 10.3389/fimmu.2021.654587, PMID: 33841438 PMC8033149

[107] Biesbroek G Kapitein B Kuipers IM Gruppen MP van Stijn D Peros TE . Inflammatory responses in SARS-CoV-2 associated Multisystem Inflammatory Syndrome and Kawasaki Disease in children: An observational study. PloS One. (2022) 17:e0266336. doi: 10.1371/journal.pone.0266336, PMID: 36449533 PMC9710748

[108] Truong DT Trachtenberg FL Hu C Pearson GD Friedman K Sabati AA . Six-month outcomes in the long-term outcomes after the multisystem inflammatory syndrome in children study. JAMA Pediatr. (2025) 179:293–301. doi: 10.1001/jamapediatrics.2024.5466, PMID: 39804656 PMC11877180

[109] Guo J Wang L . The complex landscape of immune dysregulation in multisystem inflammatory syndrome in children with COVID-19. Life Med. (2024) 3:lnae034. doi: 10.1093/lifemedi/lnae034, PMID: 39872865 PMC11749780

[110] Nakra NA Blumberg DA Herrera-Guerra A Lakshminrusimha S . Multi-system inflammatory syndrome in children (MIS-C) following SARS-coV-2 infection: review of clinical presentation, hypothetical pathogenesis, and proposed management. Children (Basel). (2020) 7:69. doi: 10.3390/children7070069, PMID: 32630212 PMC7401880

[111] Melgar M Seaby EG McArdle AJ Young CC Campbell AP Murray NL . Treatment of multisystem inflammatory syndrome in children: understanding differences in results of comparative effectiveness studies. ACR Open Rheumatol. (2022) 4:804–10. doi: 10.1002/acr2.11478, PMID: 35759535 PMC9469482

[112] Schwartz N Ratzon R Hazan I Zimmerman DR Singer SR Wasser J . Multisystemic inflammatory syndrome in children and the BNT162b2 vaccine: a nationwide cohort study. Eur J Pediatr. (2024) 183:3319–26. doi: 10.1007/s00431-024-05586-4, PMID: 38724677

[113] Le Marchand C Singson JRC Clark A Shah D Wong M Chavez S . Multisystem inflammatory syndrome in children (MIS-C) cases by vaccination status in California. Vaccine. (2025) 43:126499. doi: 10.1016/j.vaccine.2024.126499, PMID: 39515133

[114] Piechotta V Siemens W Thielemann I Toews M Koch J Vygen-Bonnet S . Safety and effectiveness of vaccines against COVID-19 in children aged 5–11 years: a systematic review and meta-analysis. Lancet Child Adolesc Health. (2023) 7:379–91. doi: 10.1016/s2352-4642(23)00078-0, PMID: 37084750 PMC10112865

[115] Roge-Gurecka I Kivite-Urtane A Pavare J . Two-year follow-up on multisystem inflammatory syndrome in children (MIS-c): findings from a tertiary paediatric hospital in Latvia. Eur J Pediatr. (2025) 184:542. doi: 10.1007/s00431-025-06253-y, PMID: 40782207 PMC12335401

